# Food fortification knowledge in women of child-bearing age at Nkowankowa township in Mopani District, Limpopo Province, South Africa

**DOI:** 10.4102/phcfm.v8i2.922

**Published:** 2016-07-29

**Authors:** Selekane A. Motadi, Vanessa Mbhatsani, Kulani O. Shilote

**Affiliations:** 1 Department of Nutrition, University of Venda, South Africa

## Abstract

**Background:**

Globally, there is evidence that three micronutrients deficiencies are of public health concern among children. They are vitamin A, iodine and iron deficiencies. Communities particularly affected are those in situations where poverty, unemployment, civil unrest, war and exploitation remain endemic. Malnutrition is an impediment to productivity, economic growth and poverty eradication. It is estimated that 32% of the global burden would be removed by eliminating malnutrition, including micronutrients deficiencies.

**Setting:**

The study was carried out in NkowaNkowa township of Mopani District, Limpopo Province, South Africa.

**Aim:**

The main objective was to determine the women’s knowledge on food fortification.

**Methods:**

The study design was descriptive. The snowballing method was used to identify women of child-bearing age. Data were collected from 120 participants using a questionnaire. The questionnaire consisted of socio-demographic, general questions on women’s knowledge on food fortification. The questionnaire was administered by the researcher using the local language Xitsonga.

**Results:**

The findings of the study revealed that a majority of 204 (57.0%) of the participants were able to define food fortification correctly while 257 (72.0%) of the participants knew which foods are fortified as well as the benefits of a food fortification programme. The majority (252 [70.0%]) of the participants knew that maize meal is one of the food vehicle used for fortification in South Africa.

**Conclusion:**

Most of the questions were answered correctly by more than 50.0% of the participants. The researcher deduced that the study participants are knowledgeable about food fortification based on the response given in relation to the programme.

## Introduction

Malnutrition is not only an urgent global health issue, but also an impediment to productivity, economic growth and poverty eradication. It is estimated that 32.0% of the global burden of disease would be removed by eliminating malnutrition, including micronutrient deficiency.^1,2^ According to Lindsay et al.,^3^ micronutrient malnutrition (MNM) is caused by monotonous diet resulting in low micronutrient intake and poor bioavailability, especially of minerals, low intake of animal-source foods, low prevalence of breastfeeding, low micronutrient density of complementary foods, increased physiological demands for growth during pregnancy and lactation, increased demand because of acute infection (especially if infection episodes are frequent), chronic infection (e.g. tuberculosis, malaria, HIV and AIDS) and disease (e.g. cancer), poor general nutritional status, in particular, protein–energy malnutrition and malabsorption because of diarrhoea.

It is also caused by the presence of intestinal parasites (e.g. *Giardia lamblia*, hookworms), increased excretion (e.g. because of schistosomiasis), seasonal variations in food availability, food shortages, social deprivation, illiteracy, low education, poor economic status and poverty.^4^ It is for this reason that in 1990, 189 United Nations’ member states committed to a set of eight Millennium Development Goals (MDGs) comprising 18 targets that were later adopted in 2000 by the United Nations as part of the Millennium Declaration. The first MDG is directly related to eradicating hunger and malnutrition. But many of the MDGs such as improving education; reducing child mortality; improving maternal health; and combating HIV and AIDS, malaria and other diseases all require good nutritional status if they are to be achieved efficiently.^5^

At the national level, nearly 4 out of 10 women knew about the food fortification legislation. This was true in all areas of residence irrespective of age of the child as well as all provinces.^6^ In 2002, the World Health Organisation^7^ report identified iodine, iron, vitamin A and zinc deficiencies as being among the world’s most serious health risk factors. Data from the study done by Labadarios et al.^6^ support these findings by highlighting that micronutrients such as zinc, iodine, vitamin A and iron remain a problem in South Africa even after fortification has been introduced. Available statistics at one of the local clinic in Nkowankowa where the researcher was based for his community nutrition practicum revealed a high prevalence of MNM-related problems among other nutrition problems affecting children under 5 years of age. This motivated the researcher to pursue a study on the knowledge of women about food fortification given the high rate of micronutrient deficiency cases in Nkowakowa township.

## Purpose of the study

The aim of this study was to assess the knowledge of food fortification in women of child-bearing age in Nkowankowa township.

## Objectives

In order to attain the aim of the study, the following objectives were developed:

- to determine the socio-demographic characteristics of women in Nkowankowa and
- to determine the women’s knowledge of food fortification.

## Methods and design

### Study design

The study design was descriptive. This study described knowledge on food fortification. A quantitative method was used to gather information. A quantitative method is a technique used to gather quantitative data or information dealing with numbers and anything that is measurable.^8,9^ A quantitative research involves the use of structured procedures and formal instruments, such as questionnaire, to collect data. To enhance objectivity, analysis of data is done using statistical procedure.

### Study population and sampling strategy

Women of child-bearing age were used. Snowballing^10^ was used where the researcher identified one woman, interviewed her and asked the interviewee to nominate another woman who had the same characteristics, for example, woman of child-bearing age and also residing in Nkowankowa township. This was done in a chain fashion until a total number of 360 participants were reached. All women of child-bearing age who could read and write in English residing in Nkowankowa township were allowed to participate. Women who were not of child-bearing age, who could not read and write in English and those not residing in Nkowankowa were excluded from the study.

### Data collection

A descriptive study was carried out on 360 women of child-bearing age. Data were collected using a questionnaire comprising the following sections: Socio-demographic information to obtain general information, questions on women’s knowledge on food fortification. The questionnaire was piloted and pre-tested to check the feasibility and understanding of the wording and phrasing of the questions. The results helped the researcher to make changes accordingly where questions were not understandable and also helped to make the questionnaire effective and refined before the survey. Permission to conduct the study in Nkowankowa township was sought from the ward counsellor. Once the counsellor granted permission, the aim and objectives of the study were explained to the participants. Interested participants were given a consent form to sign before data collection commenced. Finally, they were given a self-administered questionnaire to complete in the presence of the researcher.

### Data analysis

Data were captured in a Microsoft spreadsheet and later transferred to Statistical Package for Social Sciences (SPSS) for analysis. The SPSS version 21 was used to analyse the data. Descriptive statistics were used to interpret the socio-demographic information and knowledge of food fortification of the respondents. The average values and percentile of responses were calculated. The results are presented in figures and tables.

### Ethical considerations

Ethical clearance for the study was obtained from the University of Venda Research Ethics Committee (SHS/08/NUT/001). Permission was sought with the ward counsellor first who gave the researcher a go ahead to follow women of child-bearing age at their households. Participants were requested to complete a consent form once they showed interest to become part of the study. The researcher explained the purpose and objectives of the study. The principle of justice was adhered to through voluntary participation by study participants. In addition, anonymity and confidentiality were guaranteed by using codes instead of names.

## Results

### Age distribution of the participants

The study comprised female participants aged between 16 and 46 years. Of all the participants, 42.8% were between the age of 20 and 29 years and 36.1% were under the age of 19 years. Furthermore, 6.1% of the participants were between the age of 30 and 39 years and 15.0% were aged 40 years and above (Table 1).

**TABLE 1:** Demographics of participants (*n* = 360).

Age	Frequency	%
Under 19	130	36.1
20–29	154	42.8
30–39	22	6.1
40 and above	54	15

*Source*: Authors own work

### Location distribution

The locations studied were sections A, B and C. The participants are almost equally distributed as suggested by the percentages in the figure below. The majority (126 [35.0%]) of the participants were from Nkowankowa section C, while 120 (33.0%) were from Nkowankowa section B and 114 (32.0%) were from section A.

### Educational level and marital and employment status of women

About 66.7% of the participants had tertiary education, 22.2% had secondary education and 11.1% never attended school. The majority (50.0%) of the participants were single, 27.8% of the participants were married and 22.2% were widowed. About 44.4% of participants were employed, 26.6% were self-employed and 5.4% were unemployed (Table 2).

**TABLE 2:** The distribution of participants by highest educational level, marital and employment status.

Factor	Categories	Frequency	%
Educational level	Never been to school	40	11.1
	Primary	0	0.0
	High school	80	22.2
	Tertiary	240	66.7
Marital status	Single	180	50.0
	Married	80	27.8
	Widow	80	22.2
	Divorced	0	0.0
Employment status	Employed	160	44.4
	Self-employed	95	26.6
	Scholar	85	23.8
	Unemployed	20	5.4

*Source*: Authors own work

### Source of information

Participants were asked to select the type of media they rely on and the frequency of using it. Table 2 illustrates the distribution of participants who watched TV, read magazines and newspapers and used the Internet and radio. The results reveal that 51.4% of participants watched television in the morning, 27.8% watched it in the afternoon and 20.8% watched it in the evening. The majority (54.2%) of participants read magazine every week, 30% read it monthly and 15.8% did not read a magazines at all. About 55.0% of the participants read a newspaper every week, 1.7% read it daily and 43.9% did not read newspapers. The majority (70.8%) of the participants accessed the Internet daily, 22.2% accessed it monthly and 6.9% had no access to it. About 54.0% of the participants listened to radio in the morning, 29.4% listened to it in the evening and 16.0% listened to it in the afternoon (Table 3).

**TABLE 3:** The distribution of participants who watched TV, read magazines and newspapers, used the Internet and listened to the radio.

Source of information	*N* (%) every morning	*N* (%) every afternoon	*N* (%) every night	Daily	Weekly	Monthly	No response
TV	185 (51.4)	100 (27.8)	75 (20.8)	-	-	-	0
Magazine	-	-	-	0	195 (54.2)	108 (30)	57 (15.8)
Newspaper	-	-	-	6 (1.7)	158 (43.9)	158 (43.9)	38 (10.5)
Internet	-	-	-	255 (70.8)	0	80 (22.2)	25 (6.9)
Radio	195 (54.6)	60 (16.0)	105 (29.4)	-	-	-	-

*Source*: Authors own work

## Knowledge on food fortification

When participants were asked to list food products that are fortified in South Africa, 54.0% correctly listed maize, flour, salt and bread. Furthermore, when participants were asked about nutrients added to food, about 56.9% of participants said vitamin A, 29.2% said carbohydrates and 8.3% said minerals. Of all the participants who were asked about the diseases addressed through food fortification programme, 70.8% indicated malnutrition, 15.2% indicated HIV and AIDS and 14.0% indicated chronic diseases. In response to their knowledge about the mandatory logo stamped on fortified food packages, participants were shown various logos to choose from; 57.0% of the participants managed to choose the correct logo.

Furthermore, about 204 (57.0%) participants were able to define food fortification correctly while 257 (72.0%) participants knew which foods are fortified as well as the benefits of food fortification programme. More than half (252 [70.0%]) of the participants knew that maize meal is one of the food vehicle used in South Africa. In addition, 257 (72.0%) participants knew that children above the age of 6 months are supposed to eat fortified foods and that fortified food improves our health. In order to infer the assessment of knowledge of the study participants, the researcher reviewed the responses of participants per question and considered the majority who answered correctly as having knowledge. Most of the questions were answered correctly by more than 50.0% of the participants. The researcher deduced that the study participants are knowledgeable on food fortification (see Table 4).

**TABLE 4:** Knowledge on food fortification.

Variables	Frequency	%
**Fortified products**		
Margarine, meat, rice	30	8.3
Oil, flour, salt	30	8.3
Maize, flour, salt, bread	195	54.0
Bread, maize, salt, meat	105	29.4
**Nutrients**		
Vitamin A	205	56.9
Minerals	30	8.3
Carbohydrates	205	29.2
Protein	20	5.6
**Type of diseases addressed through food fortification**		
Malnutrition	255	70.8
HIV and AIDS	55	15.2
Chronic disease	50	14.0
**Participants responses in terms of knowing the correct logo**		
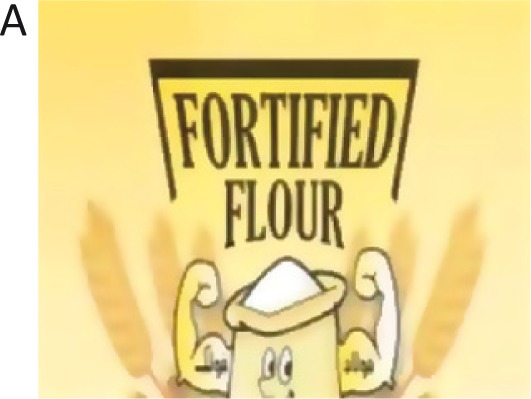	105	29.4
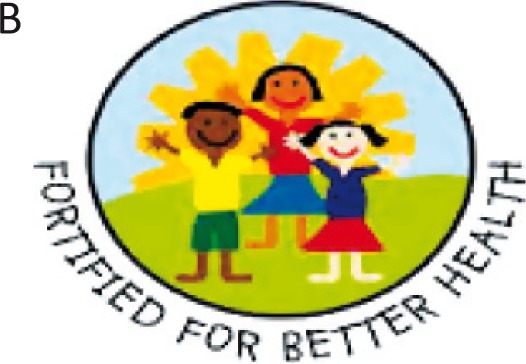	206	57.0
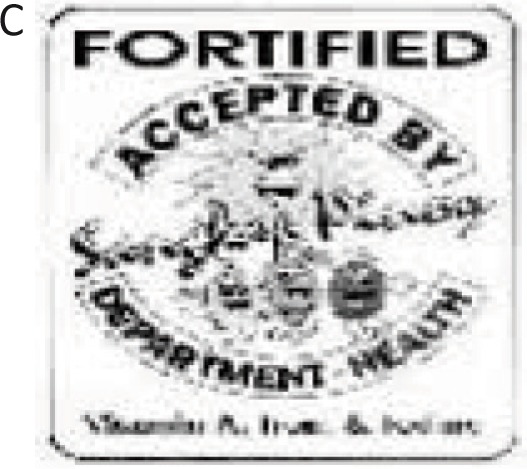	49	13.6
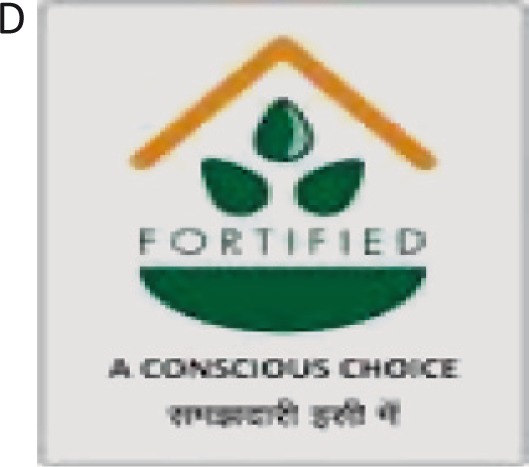	1	2.0
**General questions**		
Definition of food fortification	204	57.0
**Which are the fortified foods**		
Maize, flour, salt, bread	257	72.0
Oil, flour, salt	50	13.0
Margarine, meat, rice	53	15.0
Is maize one of the food vehicle in South Africa	252	70.0
Are children above 6 months supposed to eat fortified foods	257	72.0
Does fortified food improve our health	257	72.0
Are all types of maize meal fortified	198	55.0

*Source*: Authors own work

## Discussion

Marital status was included in the demographic data to check if single or married participants differ in terms of knowledge and our study showed that married women had more knowledge than single women. Most participants in this study (66.7%) had tertiary education and this could explain why they were knowledgeable about food fortification. Education provides knowledge and skills to encourage new behaviour and increase individual collective empowerment; it is the centre of social and economic development.^4,11^ According to Shweta,^12^ lack of knowledge of the dietary requirements and the nutritive value of different foods is an important contributory cause of widespread occurrence of malnutrition among vulnerable sections of the population in developing countries.

The study revealed that 44.4% of the participants were employed and 26.6% were self-employed and 23.8% were indicated to be students. About 15% of the employed workers were employed in financial institutions and retail shops such as fashion boutiques, supermarkets and furniture shops. About 29.4% were government employees. In contrast to UNICEF’s^13^ proportionate distribution of employed persons, our study shows that about 22.3% are paid employees, 57.2% are self-employed and 17.9% are unpaid family workers. Of those who are self-employed, women constitute about 62.0% with many of them never having attended school.

The most widely used media in this study were television, radio and newspaper. In South Africa, the Department of Health report 2004–2009 indicates that social marketing was deemed to be a critical aspect of the fortification programme. Significant funding was provided for the development of social marketing strategies and campaigns to drive consumer knowledge and demand for fortified foods. During 2000 and 2001, a broad advocacy campaign was launched through the media to sensitise and inform consumers about the prevalence and consequences of micronutrient deficiencies, the benefits of consuming fortified foods and to address any concerns they may have.^8^ The extent of the coverage in the rural areas for food fortification was regarded as an area of concern by consumer research.^14^ However, this seems to have had a positive effect for people in Nkowankowa township.

Data from the present study suggest that there is knowledge among the study participants, looking at responses of participants per question. The majority of participants know what is food fortification, how it benefits their health, age groups allowed to eat fortified food and identifying food vehicle used for food fortification. Participants having tertiary education and youth generally with high education were associated with better knowledge. Educated participants are likely to read newspaper, listen to a radio and watch current affairs on television. The majority of the study participants identified the food vehicles used for fortification. This is supported by the study done in South Africa by Labadarios et al.,^6^ where it is indicated that at the national level, nearly 4 out of 10 women knew about the food fortification legislation. This was true in all areas of residence, irrespective of the age of the participant, as well as across all provinces.

In addition, majority of the participants indicated that food fortification addresses malnutrition. Poverty, food insecurity and chronic malnutrition continue to pose significant threats to the health and well-being of South Africans, particularly women of child-bearing age and children. Malnutrition contributes up to 58% of death among children under 5 years of age.^15^ At the national level, stunting and underweight remain by far the most common nutritional disorders affecting almost 1 out of 10 children.^13^

Participants in this study managed to identify the correct logo from a list provided to them. The findings of the present study are supported by the National Food Consumption Survey study done by Labadarios et al.,^6^ where it was indicated that nationally just under one out of two women respondents included in the survey had previously seen the food fortification logo with the Eastern Cape having the lowest such exposure, but only one out of five women interpreted the logo correctly or had previously heard of the concept of food fortification. Furthermore, 8 out of 10 women nationally indicated that they would be looking for the fortification logo as well as reading the label of the food products that they buy to ensure that they were fortified. In order to sustain the elimination of MNM urged member states to include health promotion in their control strategies and this has proved beneficial in parts of South Africa. The wide use of the fortification logo in mandatory food items such as bread, flour and maize meal labels could be the main reason why participants in this study were able to identify it correctly from a list.^6^ Given the public health impact, the importance of eliminating micronutrients malnutrition and the consequences of its deficiency, it could theoretically be expected that consumers are informed and educated about food fortification.^6^ Furthermore, it has long been recognised that failure to inform and educate the public on MNM and how to address it is one of the reasons why food fortification programmes have been unsuccessful.

## Conclusion

In order to infer the assessment of knowledge of the study participants, the researcher looked at responses of participants per question and considered the majority who answered correctly as having knowledge. Participants in this study had access to various sources of information from which they were likely to hear about food fortification. These included daily access to the Internet, watching television, reading magazines and listening to the radio in descending order.

With regard to participants knowledge about food fortification, they managed to define food fortification and identify its purpose and the correct logo used in the food packages. Likewise, participants were able to identify some micronutrients of public health added in the food products and a list of food products fortified in South Africa. Most of the questions were answered correctly by more than 50% of the participants. Therefore, we conclude that the study participants are knowledgeable on food fortification based on researcher discretion of individual responses to questions asked.

## Recommendation

It is recommended that schools, health facilities, health professionals and the lay press be afforded a greater opportunity in impacting knowledge on the benefits of food fortification and fortified foods because it is evident from the present study that the source of information was mainly from media.

Notably, present efforts on the education of the public on aspects of food fortification appear to have been largely successful and should be continued and intensified. Continued and consistent message on the benefit of food fortification should be addressed to the public at large to improve on an already excellent coverage as part of the monitoring and evaluation programme on food fortification.

## References

[1] Labadarios D, Swart R, Maunder EMW, Kruger HS, Gericke GJ, Kuzawayo PMM Folate, iron, vitamin A and zinc status of children 12–108 months and women 16–35 years old in South Africa: The national food consumption survey-fortification Baseline. Nutrition Congress: Evidence based Nutrition Leading the Way in Innovation, South Africa 2008, Abstract 70; p. 202.

[2] Liyanage C, Hettiarachchi M Food fortification. Ceylon Med J. 2011;56(3):124–127. http://dx.doi.org/10.4038/cmj.v56i3.3607 22164753 10.4038/cmj.v56i3.3607

[3] Lindsay A, De Benoit B, Dary O, Hurrel R Guidelines on food fortification with micronutrients. Geneva, Switzerland; WHO Press; 2006.

[4] Lewis CJ, Crane NT, Wilson DB, Yetley EA Estimated folate intakes: Data updated to reflect food fortification, increased bioavailability, and dietary supplement use. Am J Clin Nutr. 1999;70(2):198–207. 10426695 10.1093/ajcn.70.2.198

[5] Steyn NP, Burger S, Monyeki KD, Alberts M, Nthangeni G Dietary changes and the health transition in South Africa: Implications for Health policy. Cape Town: South African Medical Research Council; 2006.

[6] Labadarios D, Steyn NP, Maunder E, et al The National Food Consumption Survey (NFCS): South Africa, 1999. Public Health Nutr. 2005;8(5):533–543. 16153334 10.1079/phn2005816

[7] World Health Organisation Obesity: Preventing and managing the global epidemic Report of a WHO Consultation on obesity, Technical Report Series No. 894 2000. 11234459

[8] Bless C, Higson-Smith C, Kagee A Fundamentals of social research methods: An African perspective. 4th ed. Cape Town: Mercury Crescent Wetteon, Juta and Company Ltd; 2006.

[9] Bowling A Research methods in health: Investigating health and health services. 2nd ed. Philadelphia, PA: Open University Press; 2002.

[10] Polit DF, Hungler BP Nursing research: Principal and method. 4th ed. Philadelphia: JB Linpincott Company; 1991.

[11] Zimmermann MB, Zeder C, Chaouki N, Saad AM, Torresani T, Hurrell RF Dual fortification of salt with iodine and microencapsulated iron: A randomized, double-blind, controlled trial in Moroccan schoolchildren. Am J Clin Nutr. 2003;77(2):425–432. 12540404 10.1093/ajcn/77.2.425

[12] Shweta T, Kumar YBN, Singh OP, Verma T, Dwivedi DK Correlation and path coefficient analysis in scented rice (Oryza sativa L.) under sodicity. Environ Ecol. 29(3B):1550–1556.

[13] UNICEF Strategies for improved nutrition of children and women in developing countries. New York: UNICEF; 1990.

[14] De Vos AS, Delport CSC, Founche CB, Strydon H Research of grass roots for the social science and him a service professional. 3rd ed. Van Schwawk Publishing Company Inc; 2011.

[15] Kinyamu HK Dietary calcium and vitamin D intake in elderly women: Effect on serum parathyroid hormone and vitamin D metabolites. Am J Clin Nutr. 1998;67(2):342–348. 9459385 10.1093/ajcn/67.2.342

